# Occurrence and Removal Characteristics of Phthalate Esters from Typical Water Sources in Northeast China

**DOI:** 10.1155/2013/419349

**Published:** 2013-03-12

**Authors:** Yu Liu, Zhonglin Chen, Jimin Shen

**Affiliations:** State Key Laboratory of Urban Water Resource and Environment, School of Municipal and Environmental Engineering, Harbin Institute of Technology, Harbin 150090, China

## Abstract

The presence of phthalate esters (PAEs) in the environment has gained a considerable attention due to their potential impacts on public health. This study reports the first data on the occurrence of 15 PAEs in the water near the Mopanshan Reservoir—the new and important water source of Harbin city in Northeast China. As drinking water is a major source for human exposure to PAEs, the fate of target PAEs in the two waterworks (Mopanshan Waterworks and Seven Waterworks) was also analyzed. The results demonstrated that the total concentrations of 15 PAEs in the water near the Mopanshan Reservoir were relatively moderate, ranging from 355.8 to 9226.5 ng/L, with the mean value of 2943.1 ng/L. DBP and DEHP dominated the PAE concentrations, which ranged from 52.5 to 4498.2 ng/L and 128.9 to 6570.9 ng/L, respectively. The occurrence and concentrations of these compounds were heavily spatially dependent. Meanwhile, the results on the waterworks samples suggested no significant differences in PAE levels with the input of the raw waters. Without effective and stable removal of PAEs after the conventional drinking water treatment in the waterworks (25.8% to 76.5%), the risks posed by PAEs through drinking water ingestion were still existing, which should be paid special attention to the source control in the Mopanshan Reservoir and some advanced treatment processes for drinking water supplies.

## 1. Introduction

Phthalate acid esters, a class of chemical compounds mainly used as plasticizers for polyvinyl chloride (PVC) or to a lesser extent other resins in different industrial activities, are ubiquitous in the environment and have evoked interest in the past decade due to endocrine disrupting effects and their potential impacts on public health [1–3].

Worldwide production of PAEs is approximately 6 million tons per year [4]. As PAEs are not chemically bound to the polymeric matrix in soft plastics, they can enter the environment by losses during manufacturing processes and by leaching or evaporating from final products [5]. Therefore, the occurrence and fate of specific PAEs in natural water environments have been observed, and also there are a lot of considerable controversies with respect to the safety of PAEs in water [5–10]. 

Six PAE compounds, including dimethyl (DMP), diethyl (DEP), dibutyl (DBP), butylbenzyl (BBP), di(2-ethylhexyl) (DEHP), and di-n-octyl phthalate (DNOP), are classified as priority pollutants by the U.S. Environmental Protection Agency (EPA). Though the toxicity of PAEs to humans has not been well documented, for some years, the Ministry of Environmental Protection in China has regulated phthalates as environmental pollutants. In addition, the standard in China concerning analytical controls on drinking waters does not specifically identify any PAEs as organic pollutant indexes to be determined by the new drinking water standard in 2007 (Standard for drinking water quality; GB5749-2006), which was forced to be monitored for the drinking water supplies in 2012. Consequently, official data about the presence of these pollutants in the aquatic environment of some cities are not available. 

Harbin, the capital of Heilongjiang Province, is a typically old industrial base and economically developed city with a population of over 3 million in Northeast China. As the newly enabled water source for Harbin city, Mopanshan waterworks supply the whole city with drinking water through long distance transfer from the Mopanshan Reservoir. To the authors' knowledge, the occurrence and fate of PAEs in the water near this area and its relative waterworks have not previously been examined. 

 The objectives of this study were (i) to determine the occurrence of PAEs and clarify the fate and distribution of the pollutants in the water source, (ii) to examine the two waterworks, where traditional drinking water treatment step was evaluated on the PAEs removal efficiencies, and (iii) to evaluate the potential for adverse effects of PAEs on human health. Therefore, the investigation of PAEs in the water can provide a valuable record of contamination in Harbin city.

## 2. Materials and Methods

### 2.1. Chemical

Fifteen PAEs standard mixture, including dimethyl phthalate, diethyl phthalate, diisobutyl phthalate, di-n-butyl phthalate, di(4-methyl-2-pentyl) phthalate, di(2-ethoxyethyl) phthalate, di-n-amyl phthalate, di-n-hexyl phthalate, butyl benzyl phthalate, di(hexyl-2-ethylhexyl) phthalate, di(2-n-butoxyethyl) phthalate, dicyclohexyl phthalate, di(2-ethylhexyl) phthalate, di-n-nonyl phthalate, di-n-octyl phthalate at 1000 *μ*g/mL each, and surrogate standards, consisting of diisophenyl phthalate, di-n-phenyl phthalate, and di-n-benzyl phthalate, in a mixture solution of 500 *μ*g/mL each, were supplied by AccuStandard Inc. As the internal standard, benzyl benzoate, was also purchased from AccuStandard Inc. All solvents (acetone, hexane, and dichloromethane) used were HPLC-grade and were purchased from J. T. Baker Co. (USA). Anhydrous sodium sulfate (Tianjin Chengguang Chemical Reagent Co., China) was cleaned at 600°C for 6 h and then kept in a desiccator before use.

### 2.2. Sample Collection and Preparation

Harbin, the capital of Heilongjiang province in China, imports nearly all of its drinking water from two sources: the Mopanshan Reservoir (long-distance transport project) and the Songhua River (old water source).

**Figure 1:**
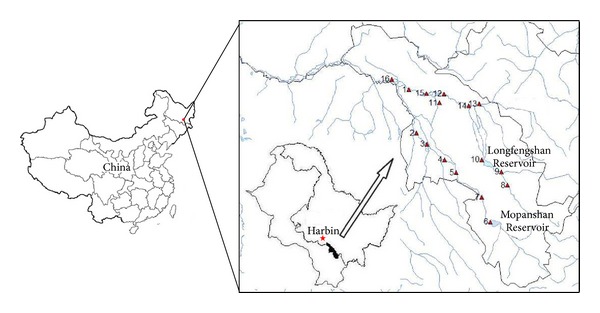
Spatial distribution of the 16 sampling sites near the Mopanshan Reservoir in Northeast China.

**Figure 2:**
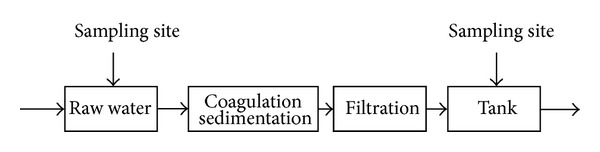
Schematic diagram of the waterworks.

Water samples from the Mopanshan Reservoir and Mopanshan waterwork were collected in 2008. The locations of the sampling sites near the Mopanshan Reservoir are presented in Figure 1; as a comparison, sampling from another Harbin water supply—Seven waterworks with water source from the Songhua River was performed in 2011. In each waterworks, with considering the hydraulic retention time, three sets of raw water samples (influent) were taken, and then the final finished waters (effluent) after the whole treatment process were carried out for the collections. The flow diagram of the waterworks is shown in Figure 2.

Samples werecollected using 2.0 L glassjars from 0.5 m below the water surface. During thewhole sampling process, the global position system was usedto locate the sampling stations (details in Table 1). All samples were transferred tothe laboratory directly after sampling and stored at 4°C prior toextraction within 2 d.

Water samples were filtered under vacuum through glass fiber filters (0.7 *μ*m pore sizes). Prior to extraction, each sample was spiked with surrogate standards. The water samples were extracted based on a classical liquid phase extraction method (USEPA, method 8061) with slight modifications. Briefly, 1 L of water samples was placed in a separating funnel and extracted by means of mechanical shaking with 150 mL dichloromethane, and then, with filtration on sodium sulfate (about 20 g), the organic extracts were concentrated using a rotary evaporator. The exchange of solvent was done by replacing dichloromethane with hexane. Finally, they were reduced to 0.5 mL under gentle nitrogen flow. The internal standard was added to the sample prior to instrumental analysis.

### 2.3. Chemical Analysis

The extracted compounds were determined by gas chromatography coupled to mass spectrometer analysis as described in other publications [11, 12]. Briefly, extracted samples were injected into an Agilent 6890 Series GC equipped with a DB-35MS capillary column (Agilent; 30 m × 0.25 mm i.d.; 0.25 *μ*m film thickness) and an Agilent 5973 MS detector, operating in the selective ion monitoring mode. The column temperature was initially set at 70°C for 1 min, then ramped at 10°C/min to 300°C and held constant for 10 min. The transfer line and the ion source temperature were maintained at 280 and 250°C, respectively. Helium was used as the carrier gas at a flow rate of 1 mL/min. The extracts (2.0 *μ*L) were injected in splitless mode with an inlet temperature of 300°C.

### 2.4. Quality Assurance and Quality Control

All glassware was properly cleaned with acetone and dichloromethane before use. Laboratory reagent and instrumental blanks were analyzed with each batch of samples to check for possible contamination and interferences. Only small levels of PAEs were found in procedural blanks in some batches, and the background subtraction was appropriately performed in the quantification of concentration in the water samples. Calibration curves were obtained from at least 3 replicate analyses of each standard solution. The surrogate standards were added to all the samples to monitor matrix effects. Recoveries of PAEs ranged from 62 to 112% in the spiked water and the surrogate recoveries were 75.1 ± 12.7% for diisophenyl phthalate, 72.3 ± 14.3% for di-n-phenyl phthalate, and 102.6 ± 10.4% for di-n-benzyl phthalate in the water samples. The determination limits ranged from 6 to 30 ng/L. A midpoint calibration check standard was injected as a check for instrumental drift in sensitivity after every 10 samples, and a pure solvent (methanol) was injected as a check for carryover of PAEs from sample to sample. All the concentrations were not corrected for the recoveries of the surrogate standards. 

## 3. Results and Discussion

### 3.1. PAEs in Water Source

**Figure 3:**
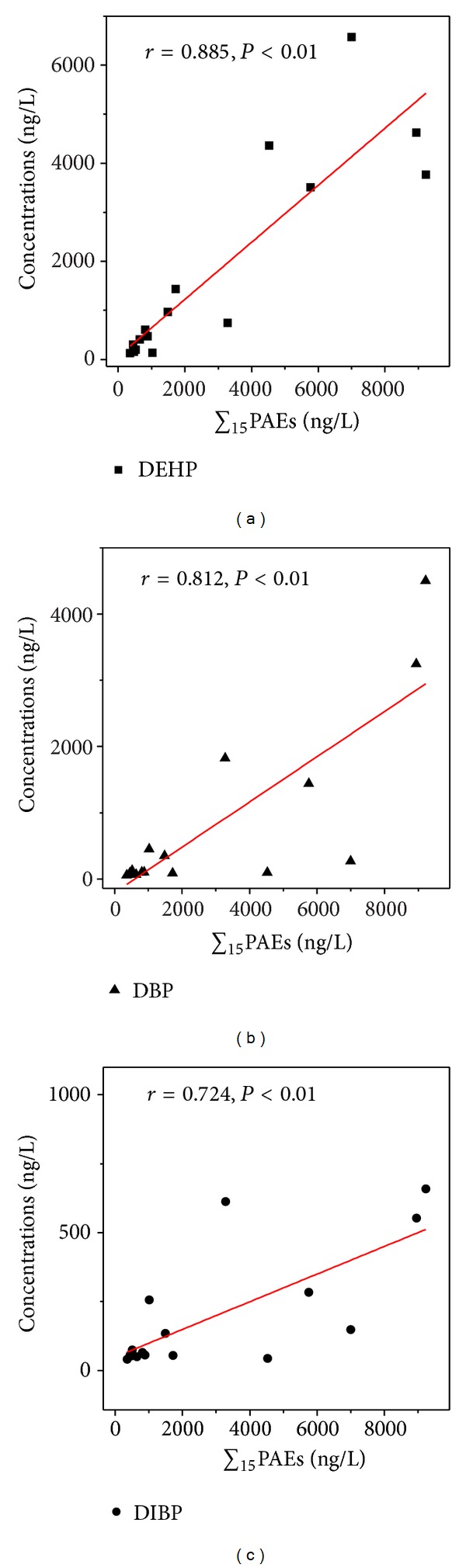
Correlations of the concentration of the major PAEs (DEHP, DBP, and DIBP) with the concentrations of total PAEs in the water samples.

The PAEs in the waters from the sampling sites near the Mopanshan Reservoir were investigated and the results are presented in Table 2. The ∑_15_PAEs concentrations ranged from 355.8 to 9226.5 ng/L, with the geometric mean value of 2943.1 ng/L. Among the 15 PAEs detected in the waters, DIBP, DBP, and DEHP were measured in all the samples, with the average concentrations being 196.6, 801.4, and 1774.1 ng/L, respectively. The three PAE congeners are important and popular addictives in many industrial products including flexible PVC materials and household products, suggesting the main source of PAE contaminants in the water [13]. The correlations of the concentration of DEHP, DBP, and DIBP with the concentrations of total PAEs in the water samples are shown in Figure 3, and a relatively significant correlation between ∑_15_PAEs and DEHP concentration was found, suggesting the important part played by DEHP in total concentrations of PAEs in the water bodies near the Mopanshan Reservoir. In other words, the contribution of DEHP to total PAEs was higher than that of other PAE congeners in the water samples. In addition, DMP, DEP and DNOP, with the mean value of 14.0, 28.4, and 54.1 ng/L, respectively, only detected at some sampling sites, and have also attracted much attention as the priority pollutants by the China National Environmental Monitoring Center. On the contrary, the concentrations of 6 PAEs (DMEP, BMPP, BBP, DBEP, DCHP, and DNP) were below detection limits in all the water samples near the Mopanshan Reservoir, which is easily explained in terms of much lower quantities of present use in China.

**Figure 4:**
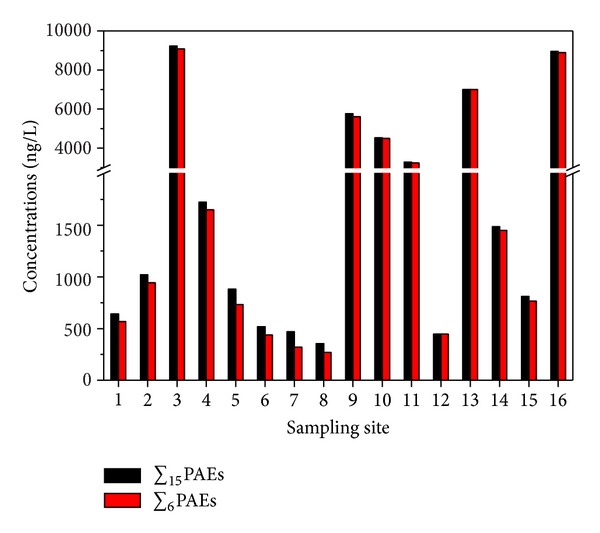
Spatial distributions of the 16 sampling sites near the Mopanshan Reservoir.

The distribution of 15 PAEs (∑_15_PAEs) studied and 6 US EPA priority PAEs (∑_6_PAEs; including DMP, DEP, DIBP, DBP, DEHP, and DNOP) in the waters from different sampling sites are shown in Figure 4. There was an obvious variation in the total ∑_15_PAEs concentrations in water samples near the Mopanshan Reservoir; the concentrations of ∑_6_PAEs from the same sampling sites varied from 269.0 to 9086.0 ng/L, with an average of 2868.6 ng/L, the distribution spectra of which observed for all the sampling sites were similar to ∑_15_PAEs. The highest levels of PAEs contamination were seen on the sampling site 3, followed by some relatively heavily polluted sites (site 16, 13, 9, 10, and 11), indicating that these sites served as important PAE sources, and the spatial distribution of PAEs was site specific. The levels in the waters examined varied over a wide range, especially in the Mangniu River near the Mopanshan Reservoir (in some case over more than one order of magnitude). In general, it should be noted that there might be a relation of PAEs levels with the input of local waste, such as sewage water, food packaging, and scrap material near the sampling point, which were found during the sampling period. 

### 3.2. PAE Congener Profiles in the Water

**Figure 5:**
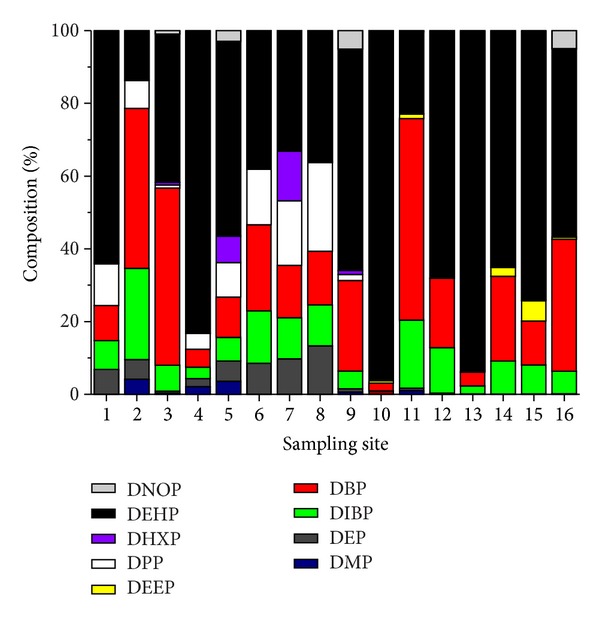
PAE composition of the water samples in 16 sampling sites.

Different PAE patterns may indicate different sources of PAEs. Measurement of the individual PAE composition is helpful to track the contaminant source and demonstrate the transport and fate of these compounds in water [11]. The relative contributions of the 9 detectable PAE congeners to the ∑_15_PAEs concentrations in the water are presented in Figure 5. It is clear that DEHP was the most abundant in the water samples with the exception of site 2, 3 and 11, contributions ranging from 33.1% to 96.3%, followed by DBP, ranging from 2.1% to 36.3%. The results are consistent with the above data for overall analysis that DBP and DEHP are the dominant components of the PAEs distribution pattern in each sampling site, which reflected the different pattern of plastic contaminant input during the sampling period. Similarly, DIBP and DBP are used in epoxy resins or special adhesive formulations, with the different proportions of these two PAE congeners, which were also the important indicator of the information polluted by PAEs for the sampling locations. Although the limited sample number draws only limited conclusions, there is still reason to note that a leaching from the plastic materials into the runoff water is possible, and that water runoff from the contaminated water is a burden pathway from different sources of PAEs [14].

### 3.3. Comparison with Other Water Bodies

Comparison with the total PAEs concentrations is nevertheless very limited due to the different analysis compounds. However, the individual PAE, namely, DBP and DEHP, is by far the most abundant in other researches, and it is possible to make a camparison with our findings. In this case, the results of DBP and DEHP concentrations published in literatures for kinds of water bodies are presented together in Table 3.

The DEHP and DBP concentrations of the present study showed, to some extent, lower concentration levels than those reported in the other water bodies in China. In comparison, the results were comparable or similar to those from the examinations described in other foreign countries. For example, as shown in Table 3, the DEHP concentrations were similar to the surface waters from the Netherlands and Italy described in the literature. Meanwhile, these concentrations in this study were quite lower than the Yangtze River and Second Songhua River in China. 

In conclusion, as compared to the results of other studies, the waters near the Mopanshan Reservoir were moderately polluted by PAEs. Therefore, there is a definite need to set up a properly planned and systematic approach to water control near the Mopanshan Reservoir.

### 3.4. PAE Levels in the Waterworks

The Mopanshan Waterworks (MPSW), equipped essentially with the water source of the Mopanshan Reservoir, has been investigated in order to assess the fate of the PAEs during the drinking water treatment process. For comparison, a sampling campaign for the determination of PAEs levels in the Seven Waterworks (SW, waterworks with the old water source of the Songhua River) was carried out. Both waterworks operate coagulation, sedimentation and followed by filtration treatment process, which are typical traditional drinking water treatment.

**Figure 6:**
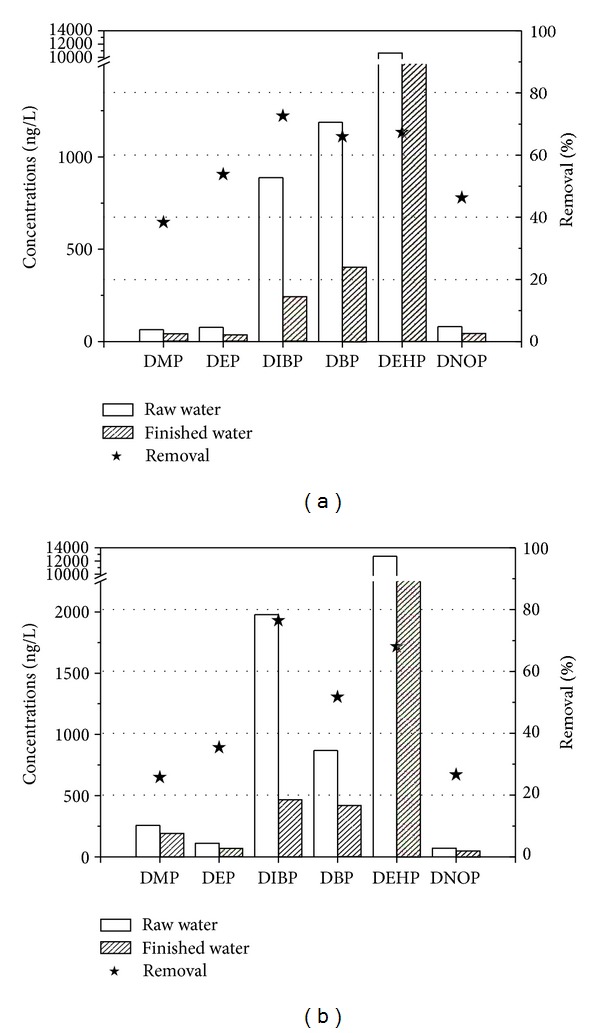
PAEs detected in the water samples from the MPSW (a) and SW (b).

The measured concentrations in the raw and finished water of the two investigated waterworks are shown in Figure 6. Six out of fifteen PAEs were detected in the finished water from the two waterworks. The detected PAEs were DMP, DEP, DIBP, DBP, DEHP, and DNOP, and the other investigated phthalates are of minor importance, with concentrations all below the limit of detection. As show in Figure 6, the measured concentrations of the analyzed PAEs in the finished water of the two waterworks varied strongly. The most important compound in the finished water was DEHP, with the mean concentration of 3473.7 and 4059.2 ng/L for the MPSW and SW, respectively, suggesting the highest relative composition of total PAE concentrations in the drinking water.

For the raw water from the different water sources, DMP and DIBP concentrations in the MPSW were much lower than the ones in the SW, and the concentrations of DEP, DBP, DEHP, and DEHP were relatively comparable in the two waterworks. The removal of PAEs by these two waterworks ranged from 25.8% to 76.5%, which varied significantly without stable removal efficiencies. The lower removal efficiencies for DMP and DNOP were observed in the SW, with the removal less than 30%. For both the waterworks, no sound removal efficiencies were obtained for the PAEs, indicating that the traditional drinking water treatment cannot show good performance to eliminate these micro pollutants, which has nothing to do with the type of the water source.

Traditional drinking water treatment focuses on dealing with the particles and colloids in terms of physical processes. Many studies of the environmental fates of PAEs have demonstrated that oxidation or microbial action is the principal mechanism for their removal in the aquatic systems [24–26]. Therefore the treatment process should be the combinations with the key techniques for removing PAEs from the water. On the other hand, since the removal efficiencies of PAEs by these advance drinking water treatments in waterworks has not been systematically studied to date, further research in this direction would seem to be required.

### 3.5. Exposure Assessment of PAEs in Water

To evaluate the potential and adverse effects of PAEs, quality guidelines for surface water and drinking water standard were used. The results indicated that the mean concentrations of DBP and DEHP at levels were well below the reference doses (RfD) regarded as unsafe by the EPA for the surface water. The levels were not also above the RfD recommended by China (Environmental Quality Standard for Surface Water of China, GB3838-2002). On the other hand, the amounts of DEHP present in public water supplies should be lower than the drinking water standard (0.006 mg/L for EPA and 0.008 mg/L for China). According to the results, the concentrations of DEHP in drinking water samples from the MPSW were lower than the limited value.

PAEs are also considered to be endocrine disrupting chemicals (EDCs), whose effects may not appear until long-term exposure. According to the results, PAEs were detected in the drinking water constantly ingested in daily life, indicating that drinking water is an important source of human exposure to PAEs contaminants. Assuming a daily water consumption rate of 2 L and an average body weight of 60 kg for adults, the average daily intake of DEHP, DBP, and DIBP by way of drinking water from MPSW was calculated to be 115.8, 13.52, and 8.1 ng/kg/d, respectively. In comparison, the values of SW with the water source of the Songhua River were relatively higher, with the calculated results of 135.3, 14.0, and 15.5 ng/kg/d for DEHP, DBP, and DIBP, respectively. In this study, the estimated daily intake levels of DEHP from the drinking water were quite lower than the RfD of 20000 ng/kg/d released by the EPA. However, some of PAEs are partly metabolised in the organism, and future experiments should be focused on determining the potential effects of the metabolites [27].

Currently treated water from the Songhua River is for nonpotable uses, and MPSW is the exclusive waterworks run for the water supply of Harbin city. However, population growth and drought cycle are limited by the availability of raw water from the Mopanshan Reservoir. To meet the increasing demand, local and regional water authorities have begun a campaign of second water supply project from the Songhua River again, which needs the protection of the Songhua River and advanced water treatment for the source.

## 4. Conclusions

This study provided the first detailed data on the contamination status of 15 PAEs in the water near the Mopanshan Reservoir. The concentration range of 15 PAEs in the samples was from 355.8 to 9226.5 ng/L, with the mean value of 2943.1 ng/L. DEHP and DBP were the main pollutants among 15 PAEs, accounting for the main watershed pollution. The occurrence and distribution of PAEs from different sampling sites in the water source varied largely, suggesting that the spatial distribution of PAEs was site specific. In addition, the monitoring of PAEs in the waterworks also showed that PAEs can be detected in the drinking water, and certain toxicological risks to drinking water consumers were found. These results reported here contribute to an understanding of how PAE contaminants are distributed in the new water source and the waterworks in Harbin city as well as forming a basis for further modeling, risk assessment, and selection of drinking water treatment technology. 

In conclusion, the results implied that no urgent remediation measures were required with respect to PAEs in the waters. However, the ecological and health effects of these substances through drinking water at the relatively lower concentrations still need further notice in light of their possible biological magnifications. Therefore, the long-term source control in the water and adding advanced treatment process for drinking water supplies should be given special attention in this area.

## Figures and Tables

**Table 1:** Detailed descriptions of the sampling locations.

Number	Sampling site	Name	Latitude	Longitude
1#	Lalin, Jinsan Bridge	Mangniu River	E127°02′53′′	N45°5′45′′
2#	Xingsheng Town, Gaojiatun	Lalin River	E127°06′11′′	N44°51′57′′
3#	Dujia Town, Shuguang	Lalin River	E127°10′44′′	N44°48′41′′
4#	Shanhe Town, Taipingchuan	Lalin River	E127°18′36′′	N44°43′50′′
5#	Xiaoyang Town, Qichuankou	Lalin River	E127°23′54′′	N44°39′46′′
6#	Shahezi Town	Mopanshan Reservoir	E127°38′59′′	N44°24′17′′
7#	Shahezi Town, Gali	Lalin River	E127°35′17′′	N44°31′56′′
8#	Chonghe Town, Changcuizi	Mangniu River	E127°46′36′′	N44°35′46′′
9#	Chonghe Town, Xingguo	Mangniu River	E127°35′17′′	N44°31′57′′
10#	Longfeng Town	Mangniu River	E127°35′19′′	N44°43′48′′
11#	Changpu Town, Zhonghua	Mangniu River	E127°16′30′′	N45°1′42′′
12#	Erhe Town, Shuanghe	Mangniu River	E127°18′32′′	N45°4′28′′
13#	Zhiguang Town, Songjiajie	Mangniu River	E127°34′3′′	N45°1′20′′
14#	Zhiguang Town, Wuxing	Mangniu River	E127°29′20′′	N45°0′43′′
15#	Changpu Town, Xingzhuang	Mangniu River	E127°10′42′′	N45°4′36′′
16#	Yingchang Town, Xingguang	Lalin River	E126°55′8′′	N45°9′0′′

**Table 2:** Concentrations of 15 PAEs in water samples near the Mopanshan Reservoir.

PAEs	Abbreviation	Water (ng/L)		
		Range	Mean	Frequency
Dimethyl phthalate	DMP	nd–42.4	14.0	8/16
Diethyl phthalate	DEP	nd–55.0	28.4	15/16
Diisobutyl phthalate	DIBP	40.0–658.8	196.6	16/16
Dibutyl phthalate	DBP	52.5–4498.2	801.4	16/16
bis(2-methoxyethyl)phthalate	DMEP	nd	nd	0/16
bis(4-methyl-2-pentyl)phthalate	BMPP	nd	nd	0/16
bis(2-ethoxyethyl)phthalate	DEEP	nd–54.6	12.8	5/16
Dipentyl phthalate	DPP	nd–92.5	45.5	10/16
Dihexyl phthalate	DHXP	nd–65.1	16.2	5/16
Benzyl butyl phthalate	BBP	nd	nd	0/16
bis(2-n-butoxyethyl)phthalate	DBEP	nd	nd	0/16
Dicyclohexyl phthalate	DCHP	nd	nd	0/16
bis(2-ethylhexyl)phthalate	DEHP	128.9–6570.9	1774.1	16/16
Di-n-octyl phthalate	DNOP	nd–448.2	54.1	5/16
Dinonyl phthalate	DNP	nd	nd	0/16
∑_15_PAEs		355.8–9226.5	2943.1	—

**Table 3:** Comparison of the concentrations of DEHP and DBP in the water bodies (ng/L).

Location	DEHP			DBP			Reference
	Range	Median	Mean	Range	Median	Mean	
Surface water, Germany	330–97800	2270	—	120–8800	500	—	[14]
Surface water, the Netherlands	nd–5000	320	—	66–3100	250	—	[15]
Seine River estuary, France	160–314	—	—	67–319	—	—	[16]
Tama River, Japan	13–3600	—	—	8–540	—	—	[17]
Velino River, Italy	nd–6400	—	—	nd–44300	—	—	[2]
Surface water, Taiwan	nd–18500	—	9300	1000–13500	—	4900	[18]
Surface water, Jiangsu, China	556–15670.7	—	—	16–5857.5	—	—	[19]
Yangtze River, mainstream, China	3900–54730	—	—	nd–35650	—	—	[20]
Middle and lower Yellow River, China	347–31800	—	—	nd–26000	—	—	[21]
Second Songhua River, China	nd–1752650	370020	—	nd–5616800	—	717240	[22]
Urban lakes, Guangzhou, China	87–630	170	240	940–3600	1990	2030	[11]
Xiangjiang River, China	620–15230	—	—	—	—	—	[23]
This study	128.9–6570.9	671.0	1774.1	52.5–4498.2	110.3	801.4	
